# Male Fertility and Internal Migration in Rural and Urban Sub-Saharan Africa

**DOI:** 10.1007/s10680-023-09659-2

**Published:** 2023-03-28

**Authors:** Ashira Menashe-Oren, David A. Sánchez-Páez

**Affiliations:** Centre for Demographic Research (DEMO), Université Catholique de Louvain, Place Montesquieu 1, 1348 Louvain-La-Neuve, Belgium

**Keywords:** Male fertility, Internal migration, Sub-Saharan Africa, Rural, Urban

## Abstract

Subnational differences in male fertility within sub-Saharan African countries have not been explored, nor the differences in male fertility according to migration status been sufficiently probed. We study divergences in rural and urban male fertility and investigate the relationship between male fertility and migration across 30 sub-Saharan African countries. We employ 67 Demographic and Health Surveys to estimate completed cohort fertility among men aged 50–64 according to migration status. Overall, we find that urban male fertility has declined faster than rural male fertility, widening the gap between the sectors. Rural-urban migrant men have lower fertility than their rural non-migrant counterparts. Men migrating within the rural sector have similarly high fertility as rural non-migrants, while urban–urban migrant men have even lower fertility than non-migrant urban men. Using country-fixed effects models, we find that among men with at least secondary education, differences in completed cohort fertility by migration status are widest. When we consider the timing of migration in relation to the timing of the birth of the last child, we observe that migrant men are a select group, having around two children less than non-migrant rural men. There is also evidence of adaptation to destination, though to a lesser extent. Furthermore, migration within the rural sector does not seem to be disruptive to fathering. These results indicate that rural-to-urban migration has the potential to delay rural fertility decline, and that urban male fertility is likely to decline further, especially as the proportion of urban-to-urban migration increases.

## Introduction

Fertility remains high in sub-Saharan Africa (SSA), with significant differences between urban and rural areas (Corker, 2017; Lerch, 2018, 2019; Schoumaker & Sánchez-Páez, 2020; Shapiro & Gebreselassie, 2008; Shapiro & Tambashe, 1999). According to measures of female fertility, fertility first declines in urban areas (Lerch, 2018; Shapiro & Gebreselassie, 2008), and the pace of decline in the rural sector is slower (Lerch, 2019). More recent evidence suggests that fertility is stalling in urban SSA (Schoumaker & Sánchez-Páez, 2020).

Yet, to our knowledge, no research has examined within-country variance in male fertility in SSA. Fertility differences between the rural and urban sectors are expected as each sector progresses through the demographic transition at different paces (Dyson, 2011). The levels of fertility in each sub-national area are further compounded by the movement of people from one area to the other. Indeed, high rural fertility creates a large pool of potential migrants to the urban sector. Since migrants to the urban sector are largely of reproductive ages (Bernard et al., 2014; Menashe-Oren & Stecklov, 2018; Montgomery et al., 2003; Rogers et al., 2002), they also drive higher fertility in urban areas. Distinguishing rural and urban fertility is further complicated by high levels of circular migration in SSA (Beguy et al., 2010; Potts, 2009).

Both migration and fertility are important events over the life-course. Although previous research has examined the relationship between female migration and fertility (Brockerhoff, 1996; Chattopadhyay et al., 2006; Goldstein, 1973; Jensen & Ahlburg, 2004; Lee & Pol, 1993), few studies have looked at male fertility and migration. Exceptionally, Cantalini and Panichella (2019) examined international migration in Europe, finding immigrant men to have more children. Within SSA, Pongi Nyuba (2019) found that in rural Burkina Faso internal migrant men have similarly high fertility to migrant women. Moreover, while male migrants to informal settlements of the capital city have lower fertility, internal migrants to formal settlements tend to adapt to destination fertility levels. These unique studies are limited in scope, the first addressing international migration in Europe, and the second, only one country. We propose a wider perspective, covering multiple countries in SSA. Additionally, we focus on internal migration in particular since it comprises the majority of movements in SSA (Abel & Sander, 2014; Adepoju, 1998; Deshingkar & Grimm, 2005; King & Skeldon, 2010; United Nations, 2009), and it has the potential to further diverge urban and rural fertility patterns.

There are differences in both fertility levels and in migration patterns between men and women (Bernard et al., 2014; Schoumaker, 2019). We expect that migration would differentially affect male fertility. Studies suggest that male fertility is higher than female fertility in SSA because of the age difference between spouses, their differential mortality, and the potential of having children from multiple partners—even simultaneously (Field et al., 2016; Schoumaker, 2019). Moreover, polygyny is common in SSA (Timæus & Reynar, 1998) and drives a larger gap between male and female fertility (Field et al., 2016; Schoumaker, 2019). Indeed, polygynous settings are associated with social inequalities, reinforcing gender norms around reproductive decision-making (Agadjanian & Ezeh, 2000; Smith-Greenaway & Trinitapoli, 2014). Furthermore, the reproductive period among men is longer, and harder to delimit by age, leaving a greater period of years of fatherhood and, consequently, it is possible to have children before, during and after migration, even at ages above 45 years. The motivation and determinants of having children may also differ between men and women, with men often desiring larger families (Doepke & Tertilt, 2018).

Similarly, migration patterns in SSA differ by sex, with working-aged men generally migrating within countries at higher rates than women (Menashe-Oren & Stecklov, 2018). Notably, women tend to migrate at younger ages, peaking at around ages 15–19 (Beauchemin, 2011; Menashe-Oren & Stecklov, 2018; Bocquier et al., 2023) for education, marriage and family reunification, while traditionally men migrate in older ages mostly for work (Coulter & Scott, 2015; Duncan & Perrucci, 1976; Fleury, 2016; Geist & McManus, 2012; Thomas, 2019).

### The Relationship Between Internal Migration and Fertility

Four mechanisms have been explored to explain the relationship between female fertility and migration. The first, socialisation, suggests that the place of origin has the greatest impact on women, and a migrant will maintain fertility behaviour as in their origin (Goldberg, 1959; Hervitz, 1985). The second, selection, suggests that migrants are a select group (with different characteristics) which explains why they have different fertility to both origin and destination populations. Evidence of selectivity of migrants in SSA has been found among both rural-to-urban and urban-to-rural migrants (Chattopadhyay et al., 2006). The third, adaptation (sometimes referred to as assimilation), implies that the longer a migrant is in new location the more likely they are to adapt to fertility norms at destination. Jensen and Ahlburg (2004) document adaptation in the Philippines where fertility is lower among migrant women when they are employed at urban destinations. And while controlling for selection, adaptation among rural-to-urban migrants has a powerful effect on fertility reduction in Ghana, Mexico and Korea (though not in Cameroon) partly due to effective urban family planning (Gyimah, 2006; Lee & Pol, 1993). Finally, disruption implies that fertility is delayed due to partner separation or physical stress. Evidence of disruption has been documented among rural-to-urban migrants in Brazil, Malaysia and Thailand (Bach, 1981; Goldstein, 1973; Hervitz, 1985).

These mechanisms of socialisation, selection, disruption and adaptation are generally assumed to apply to men too, though scantly investigated empirically. In one inquiry, disruption around the time of moving among international migrant men in European countries was found to temporarily lower male fertility, while socialisation appeared to kick in in the long run especially among men from Africa, Middle East and Asia, increasing destination fertility (Cantalini & Panichella, 2019). In another study focusing on Western Africa, men migrating to cities were found to delay entry into parenthood (disruptive effects), and selection of men was found to be an important factor among prospective migrants (Pongi Nyuba, 2019).

The four mechanisms have been used to explain fertility trends among both internal and international migrants. However, most of these mechanisms are likely weaker when examining migration within the same area, rural-to-rural or urban-to-urban migration, since the environments are similar, and fertility levels comparable. Notably, socialisation and adaptation are irrelevant for intra-sector migration flows, while disruption effects may be moderated, as familiarity with the lifestyle may make moving easier. Selection of migrants, with different characteristics to non-migrants, could be the main mechanism influencing fertility among intra-urban or intra-rural migrants.

These four mechanisms attempt to simplify a much more complex relationship between fertility and migration. Reasons for migration may be important to consider; for example, refugees may suffer from disruption to a greater extent, or migration for marriage would clearly encourage childbearing. Moreover, even when an individual does not migrate, their partner may migrate and affect their fertility. Men who migrate unaccompanied by their partners have been found to lower women’s fertility (Agadjanian et al., 2011; Yabiku et al., 2010). In contrast, when migration is economically beneficial, larger families may be desired (Agadjanian et al., 2011; Omondi & Ayiemba, 2003). Indeed, male fertility tends to be higher when they are employed, and socio-economic conditions are good (Tragaki & Bagavos, 2014). Women whose partners migrate may also be exposed through their partner to different ideas of reproductive norms, derived from the cultural environment at migrant destination. This sort of diffusion may include use of modern contraceptives or investment in quality education (Beine et al., 2013; Bertoli & Marchetta, 2015; Montgomery & Casterline, 1993).

The mechanisms are further complicated by multiple moves, circular migration, and with whom individuals migrate. Migrants who move as a couple (or family) have higher fertility than independent or first-time migrants (Ortensi, 2015). Moreover, a migrant who divides time between two or more areas annually will constantly have forces of adaptation and disruption impacting their fertility (assuming that during childhood they remained in one location and were socialised accordingly). Likewise, the selection (characteristics) of such a migrant would be different to the selection of a one-time migrant.

Fertility may have an effect on migration too (Pongi Nyuba, 2019). On the one hand, individuals with no children, or with up to two children, are more flexible and likely to migrate, due to the costs associated with migration (Brockerhoff & Eu, 1993). On the other hand, (multiple) children may also drive migration when seeking to live in settings more suited to child-raising, such as places with better schools (Kulu, 2008). In some agricultural settings, having more children allows families to send some of them to cities from where they can eventually increase the family capital (Basu, 1999).

In light of the relationship explored between female fertility and migration, we propose a first cross-country investigation of male fertility and internal migration. In this study, we start by outlining rural and urban male fertility differences in SSA. We examine whether migrant men between rural and urban areas have different fertility in comparison with their origin and destination. We further address whether these differences are maintained once we account for education, and whether these differences are likely due to adaptation or selection.

## Data and Methods

### Measuring Male Fertility

Studies have traditionally examined the link between fertility and migration in SSA countries mainly for women due to the availability of data. Unfortunately, men’s data is scarce although some surveys have started including them. The Demographic and Health Surveys (DHS) remain the main source of information on fertility in SSA countries for both men and women. The DHS collect vast amounts of information from women aged 15–49 on fertility and migration, such as birth histories, place of current residence, place at birth, and time in the new location. From men, such information is mostly collected for the age group 15–64, although in less detail as for women. For instance, men are only asked about the number of children ever born and in some cases the age of the youngest child. Despite this data limitation we are able to estimate a cohort measure of fertility for our main analysis.

From the available data in the DHS male records, we compute completed cohort fertility (CCF), which is the arithmetic mean of the values of completed fertility for all individuals in the cohort of 50–64 years old. In computing the CCF, we use the sample weights calculated in the DHS. CCF is a useful measure of fertility since cohort measures are less volatile than period measures, like total fertility rates (TFR),^1^ and are not affected by shocks occurring in the year in which they are measured. Moreover, CCF is a favourable measure of fertility since it will no longer vary after measurement, meaning that it can reflect the total effect of migration on fertility levels. Using CCF also allows us to examine the changes in rural and urban fertility in SSA over three decades. All the same, by using CCF, we can only measure fertility among men who have already finished having children. The implication of this is that more recent trends in rural and urban fertility, or in a potentially dynamic relationship between migration and fertility are not captured. Moreover, fewer men in older ages are interviewed in the DHS, and Also, men’s reports on their children may be inaccurate. In surveys, men tend not to report on nonmarital births (Joyner et al., 2012). Also, although it is unlikely, there is a possibility that some men may not know they have any additional children.

We limit our analysis to cohorts of men who are over age 50 and above for two reasons. First, we do not want to further reduce the sample size by only examining men aged 60 and above, due to the limitations of the upper boundary of the age of men interviewed by the DHS (age 64), and the overall low proportion of men interviewed over age 50. We test for sensitivity of using this lower bound of 50 years old by extending the age group to a lower bound of 45 years and thus increasing the sample size. Second, although biologically men can continue to have children into older ages, the proportion of men who do have children between ages 50 and 60 in SSA has been shown to be small (Field et al., 2016). Thus, they have finished, or are close to finishing having children. From our sample, on average only 4.8% of men over 50 years old have partners who were pregnant at time of the survey, though this differs by survey, with a maximum of 12.8% in Mali and 11% in Burkina Faso or Chad. Moreover, only 11.1% of men, excluding those with partners who are already pregnant, report wanting to have another child. Our CCF estimates are thus likely biased downwards, particularly in Sahelian countries where the average age at fatherhood is over 40 years old (Schoumaker, 2019). Overall, we are cautious in our CCF estimates and emphasise that they are likely underestimates of fertility, particularly in polygynous societies.

### Defining Migration Status

We define migrants as men who currently live in a different rural/urban area as compared to previous residence and who moved there after their 15th birthday. We use this cut-off age assuming that migration in younger ages is associated with faster adaptation. So, we consider men who migrated as children as non-migrants. To identify migrants, we combined two questions from the DHS. The first, current place of residence is either urban or rural. The second question is on the previous place of residence. In this case, there are five options: capital or large city, city, town, countryside and abroad. We define as “urban non-migrants” those currently living in urban areas and always having lived there. “Rural non-migrants” are those currently living in rural areas and always lived there. “Rural-to-urban migrants” are those currently living in urban areas but lived previously in the countryside. “Urban-to-rural migrants” are those currently living in rural areas but living previously in capital or large city, city or town. Those who migrated within rural areas (rural–rural) or within urban areas (urban–urban) are within-sector migrants. We examine ever-migrants in these six categories and exclude from our analysis international migrants. Considering the small sample size within each category of migrants, we test for sensitivity of some of our results by only considering surveys with at least 30 men in each migrant status.

We do not limit migrant status by time, including recent migrants and past migrants in the same group. On average, urban-to-rural migrants migrated 15.6 years ago (standard deviation (SD) = 10.5 years), rural-to-urban migrants migrated 18.9 years ago (SD = 11.3), urban-to-urban migrants, 15.4 years ago (SD = 10.9) and rural-to-rural migrants, 16 years ago (SD = 10.8). We acknowledge that this does not consider the stages of adaptation, with the longer the duration of stay in the sector, the closer in behaviour we would expect the migrant to be to the destination fertility. However, considering the relatively small sample of men for whom we can estimate CCF, if we were to consider only recent migrants we would not be able to model male fertility. All the same, we are able to partially account for the timing of migration by considering whether it was before or after the birth of the last child. We also include a measure of duration in current residence in this model. This allows us to unpack whether migrant fertility is different because of selection or adaptation effects.

### Estimates from Pooled DHS

We select our data based on all available DHS which include men of ages 50 and above, and the variables which allow us to identify migration. As a result, we include in our analysis 67 surveys covering 30 SSA countries. Table 1 summarises the countries and periods covered, the proportion of migrants, and the estimated national-level CCF. Half of the surveys were conducted between 2000 and 2009, and for nine of the countries, we only have one survey. In the vast majority of surveys, non-migrant rural men compromise the greater part of all men, and migrants within sectors outnumber migrants between sectors. In Gabon there is a high proportion of within-urban migration, a reflection of the high percent of the population living in the urban sector (79% at time of the survey). In some countries, like Ghana and Cameroon, migration to the rural sector is common. This may be capturing circular migration trends, or return migration in old ages (Clark et al., 2007; Levira et al., 2014). In our sample, about 60.5% of urban-to-rural migrants report that their childhood place of residence was the countryside, indicating that they are mostly return migrants. Even in younger ages, urban-to-rural migration in SSA is common (Beauchemin, 2011; Beauchemin & Bocquier, 2004; Potts, 1995), often leading to zero net migration (Bocquier et al., 2023).

**Table 1:** Description of fertility and migration based on the DHS data used (population weighted)

	Men aged 50–64							Completed cohort fertility
	Total	% Non-migrants		% Migrants				
				Between areas		Within areas		
		Urban	Rural	To rural	To urban	To urban	To rural	
*Benin*								
1996	203	13.8	49.8	8.4	3.4	14.3	10.3	11.9
2001	212	12.3	44.8	9.9	6.1	16.0	10.8	11.6
2006	569	16.2	46.0	11.1	6.2	14.4	6.2	10.9
2017	792	27.4	45.1	7.1	3.9	12.5	3.9	9.4
*Burkina Faso*								
2003	394	5.8	52.5	11.2	2.0	6.6	21.6	11.1
*Burundi*								
2016	864	2.8	70.0	20.7	0.0	6.5	0.0	7.8
*Cameroon*								
1998	209	6.7	43.1	19.6	6.7	12.4	11.5	7.9
2018	846	18.3	27.8	18.8	4.6	23.6	6.7	7.8
*Central African Republic*								
1994	180	13.3	35.0	17.8	6.7	15.6	11.1	7.8
*Chad*								
1996	162	6.2	58.0	6.8	8.0	7.4	14.2	11.2
*Democratic Republic of Congo*								
2007	427	15	32.6	10.8	11.2	9.6	20.8	8.8
*Ethiopia*								
2000	283	3.2	75.6	4.2	1.4	3.9	11.7	8.3
2005	559	2.7	78.0	2.0	4.7	3.2	9.7	8.2
2016	1081	6.7	66.4	15.4	0.0	11.5	0.0	7.7
*Gabon*								
2000	135	12.6	10.4	17.8	5.9	42.2	11.1	7.8
*Ghana*								
1993	160	1.9	15.6	32.5	3.8	26.9	19.4	7.5
1998	161	5.6	32.9	21.7	5.0	21.7	13.0	7.8
2003	482	6.6	21.6	22.0	8.1	26.8	15.1	7.4
2008	501	10.8	24.0	16.8	5.6	34.7	8.2	6.2
*Guinea*								
2005	464	5.2	50.0	9.7	7.8	15.1	12.3	9.4
2018	505	19	60.2	7.7	1.4	8.9	2.8	8.0
*Kenya*								
1998	181	2.8	47.5	20.4	11.6	12.7	5.0	8.4
2003	213	4.2	31.9	18.8	5.6	14.1	25.8	7.6
2008	206	2.9	54.4	7.8	3.9	10.7	20.9	6.7
2014	634	9.3	41.2	6.8	12.3	8.4	22.1	6.4
*Lesotho*								
2004	292	7.5	61.6	4.5	6.5	2.1	17.8	5.2
2009	307	3.9	47.9	9.4	12.4	10.4	16.0	4.3
*Liberia*								
2019	428	27.8	36.2	13.8	4.4	15.4	2.3	7.6
*Madagascar*								
2003	216	13.0	43.5	15.3	2.8	10.6	14.4	6.1
2008	932	8.0	63.4	7.2	2.1	6.7	12.6	6.6
*Malawi*								
2000	176	1.1	39.2	9.7	3.4	4.0	42.6	9.3
2004	148	0.7	37.2	8.1	2.0	2.7	49.3	8.7
2010	356	3.7	40.2	7.0	4.8	7.6	36.8	7.8
2015	350	4.0	51.7	9.7	0.9	7.1	26.3	7.2
*Mali*								
1995	299	7.7	37.8	23.4	12.7	8.7	9.4	10.3
2001	328	6.4	48.8	15.2	6.7	8.8	13.7	9.7
2006	497	14.3	47.1	16.5	6.4	7.8	7.6	10.0
2018	588	8.3	63.6	10.0	2.0	7.1	8.8	8.9
*Mozambique*								
1997	356	3.7	61.8	9.0	3.9	5.6	16.0	7.9
2003	408	12.7	52.0	4.4	6.6	11.8	12.5	8.4
*Namibia*								
2000	183	16.9	33.9	6.6	13.7	12.0	16.9	7.1
*Niger*								
1998	388	5.4	65.7	2.6	5.7	5.2	15.5	10.8
2006	435	7.8	69.4	4.6	4.6	6.9	6.9	10.7
*Nigeria*								
2003	252	10.7	38.9	12.7	6.0	15.1	16.7	9.5
2008	1657	12.0	46.0	11.6	2.7	18.7	8.9	8.9
2018	1442	22.9	42.9	6.5	3.0	20.6	4.2	8.5
*Rwanda*								
2000	184	4.9	64.1	1.1	5.4	6.5	17.9	8.8
2005	405	6.7	57.3	1.2	2.7	4.2	27.9	8.9
2019	666	3.0	51.7	3.5	5.4	8.1	28.4	6.5
*Senegal*								
2005	303	15.2	26.7	10.6	7.6	22.8	16.8	9.4
*Sierra Leone*								
2008	333	11.1	48.6	9.3	3.6	13.5	13.5	8.7
2019	812	10.5	39.3	15.9	4.6	19.3	10.2	7.8
*South Africa*								
2016	390	18.2	17.4	4.9	14.1	38.7	6.7	3.8
*Tanzania*								
1991	273	13.2	42.1	6.6	5.5	7.0	25.6	8.7
1996	215	5.1	64.2	6.0	3.7	4.2	16.7	9.0
*Togo*								
1998	221	13.1	39.8	11.3	4.1	7.2	24.4	10.2
*Uganda*								
1995	93	1.1	47.3	9.7	1.1	3.2	36.6	8.5
2006	116	0.9	38.8	5.2	3.4	3.4	48.3	10.1
2016	299	6.7	42.8	5.0	4.3	8.0	33.4	9.2
*Zambia*								
1996	115	8.7	16.5	18.3	9.6	19.1	27.8	9.5
2001	169	7.1	21.3	16.6	7.1	21.3	26.0	9.6
2007	496	7.3	19.6	14.7	3.6	29.8	25.2	8.2
2013	1189	9.8	26.3	12.1	4.6	24.6	22.5	8.4
2018	951	7.4	29.5	12.2	8.3	19.1	23.4	7.6
*Zimbabwe*								
1999	101	8.9	27.7	3.0	12.9	13.9	32.7	7.5
2005	304	22.4	38.2	4.9	10.2	10.5	13.8	6.8
2015	352	9.1	30.7	33.8	0.0	26.4	0.0	5.6
Total	28,448	10.5	45.3	11.2	4.9	14.2	13.8	8.2

Using the most recent survey for each country, the lowest CCFs are recorded in Lesotho in 2009 (4.3 children per men) and South Africa in 2016 (3.8), while the highest CCFs are found in Niger in 2006 (10.7), Burkina Faso in 2003 (11.1) and Chad in 1996 (11.2). In countries with multiple DHS surveys, we note a general decline in CCF over time. These differences across countries and over time are in line with female fertility trends (United Nations, 2019).

To conduct our analyses, we first examine changes over time of completed fertility by rural/urban area of residence at time of the survey. To do so, we pool all DHS to compute CCF by place of residence and use cubic splines to smooth the trends. Second, we analyse the difference in levels of CCF by migration status according to place of origin and destination. We compare these results with those of TFR measured among men aged 15–64 years old, using the date of birth of the last-born child (Schoumaker, 2019), for a subset of surveys. Third, we propose a series of ordinary least square (OLS) regressions to model the number of children ever born to men aged 50–64. We first estimate a model that only includes current place of residence to explore the differences between urban and rural areas. Then, we estimate a baseline model, which accounts for migration status and the number of women with whom a man has fathered children to control for high levels of male fertility due to polygamy or re-partnering. We next include educational attainment, which has been found to be an important determinant of both migration and fertility by place of residence (Dustmann & Glitz, 2011; Shapiro & Tenikue, 2017). We do not include wealth as an indicator of socio-economic status in our models since wealth is measured at the time of the survey and it is collected at household level and is missing in many DHS. We also note that wealth is highly correlated with education. We estimate a further model including the interaction of migration with education to explore the education-gradients of fertility and migration. Finally, we model the number of children ever fathered while differentiating between migration before or after the birth of the last child. This model allows us to explore whether there are some adaptation or selection effects. Importantly, this model excludes childless men, who overall make up 2% of our original sample,^2^ which is slightly lower than the 3.5% of men of over age 40 across sub-Saharan Africa (Verkroost & Monden, 2022). Childlessness among rural-to-urban migrant men is 2.4%, while it is slightly lower among urban-to-rural migrant men (1.6%). Within regions, childlessness among rural-to-rural migrant men is higher (1.9%) than among urban-to-urban migrants (1.6%). Childlessness is slightly lower among rural non-migrants (2.1%) than among urban non-migrants (2.3%). All models include fixed effects at the country-level, to control for unobserved heterogeneity, and year of the survey to control for changes in fertility levels over time. All models use the sample weights computed in the DHS.

## Results

### Urban Male Fertility Has Declined, and is Lower than Rural Fertility

**Fig. 1:**
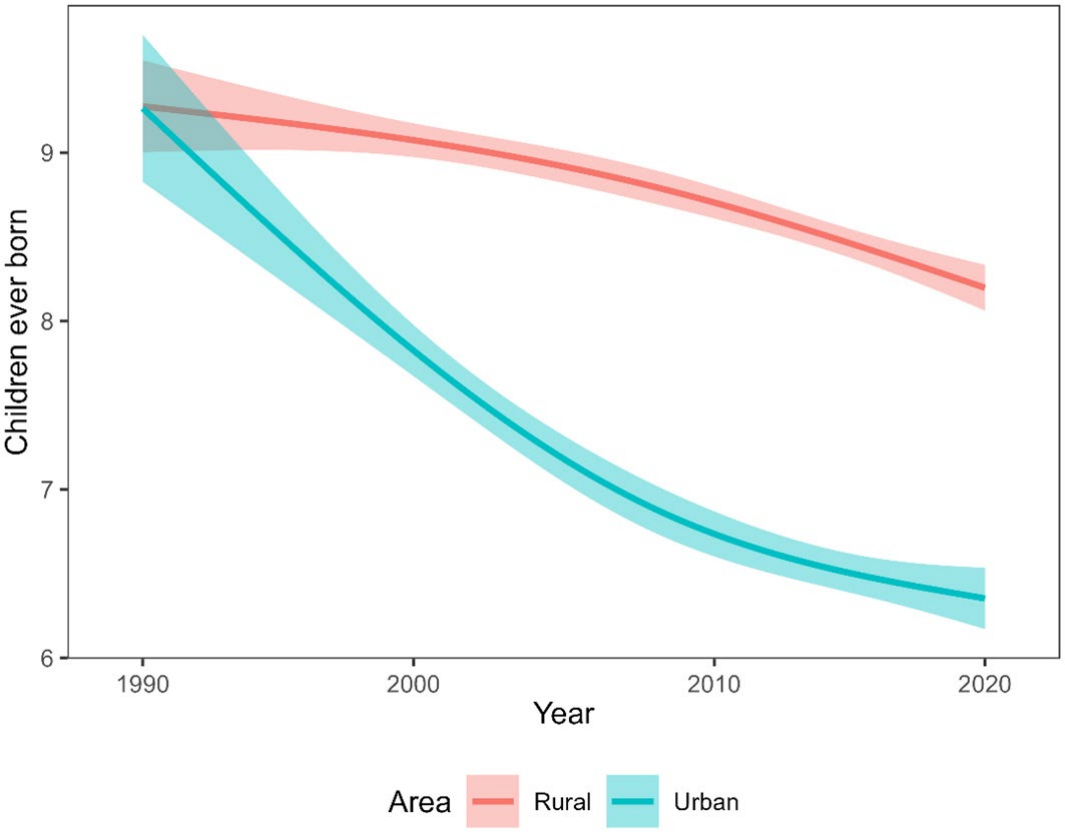
Linear-fitted rural and urban male fertility trends over time, based on completed fertility of 50–65-year-old men according to men’s current place of residence, with 95% confidence intervals. *Note*: Rural and urban fertility is determined by men’s place of residence at time of survey

Figure 1 displays the trends of male completed fertility by place of residence at time of survey, over time. Overall, we note a widening of the gap between the areas, with urban fertility declining faster than rural. Urban cohort fertility decreased from around nine children ever born in the early 1990s to slightly above six in recent years. Over the same period, rural fertility declined on average by 1.5 children from 9.4. The slow pace of decline in the rural area could be explained by higher rural polygyny prevalence (Timæus & Reynar, 1998). While Fig. 1 indicates the year of the survey, it is important to note that the CCF are comparable to earlier levels of total fertility rates (TFR)—approximately forty years before,^3^ considering the middle of the reproductive-fatherhood period of men (Schoumaker, 2019).

**Fig. 2:**
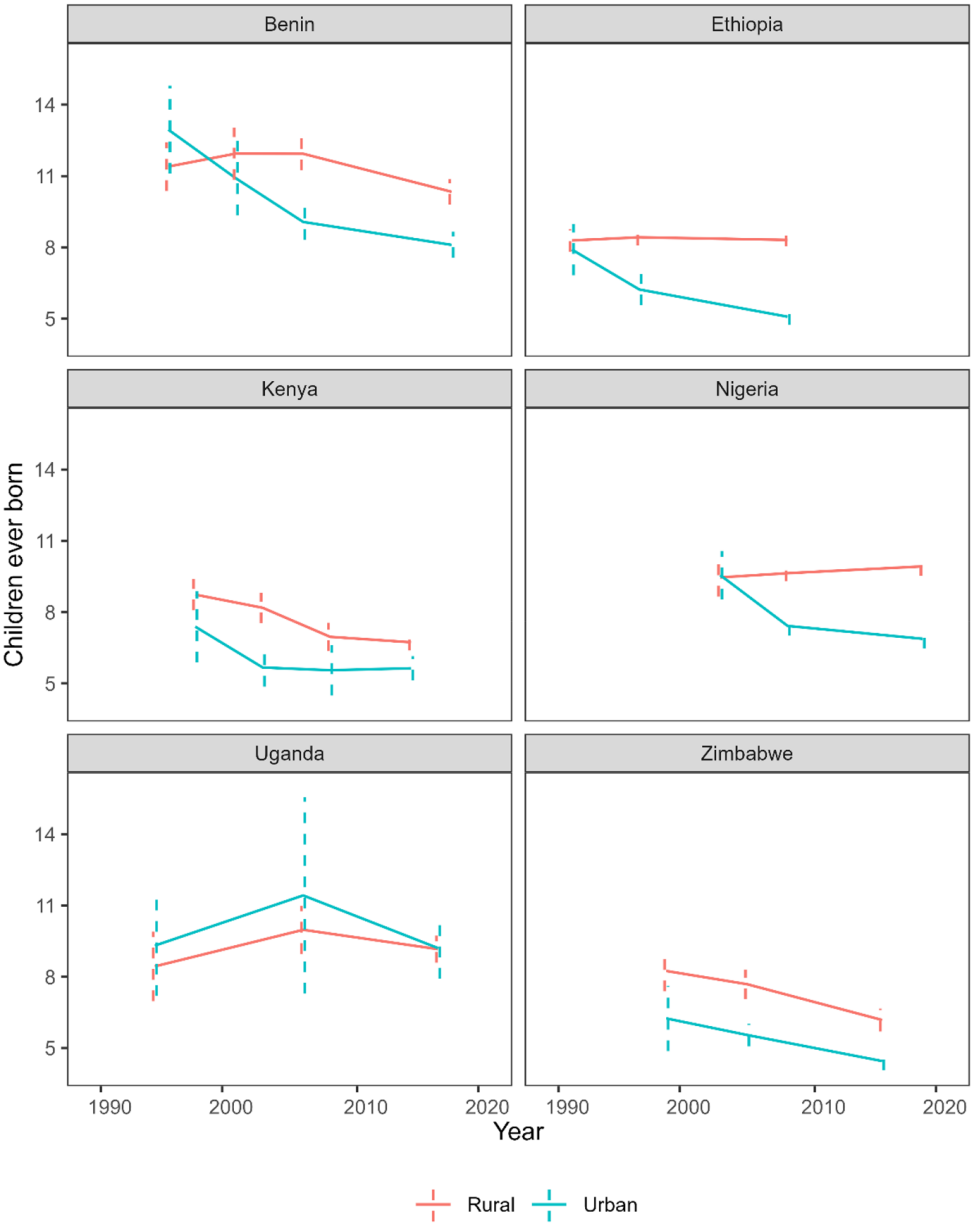
Rural and urban male fertility trends over time in selected countries with at least three DHS, based on completed fertility of 50–64-year-old men according to men’s current place of residence, with 95% confidence intervals. *Note*: Rural and urban fertility is determined by men’s place of residence at time of survey

Since Fig. 1 conceals considerable heterogeneity within SSA, in Fig. 2, we further explore the rural/urban fertility trends within selected countries. In most countries, CCF declined significantly in urban areas over the last 30 years, while in rural areas, we find modest declines or even increased fertility. Uganda is the only country where urban male completed fertility is higher than rural CCF across surveys, though the fertility levels are relatively similar between areas, and confidence intervals overlap. In Benin, the earliest survey also suggests higher urban fertility, but thereafter rural fertility is higher and the gap between areas widens. In rural Ethiopia and rural Nigeria, CCF remained high while urban CCF declined. In Kenya and Zimbabwe, a similar rural/urban gap in fertility is maintained over time. The remaining countries with at least two DHS available are included in Appendix Fig. 6.

**Fig. 3:**
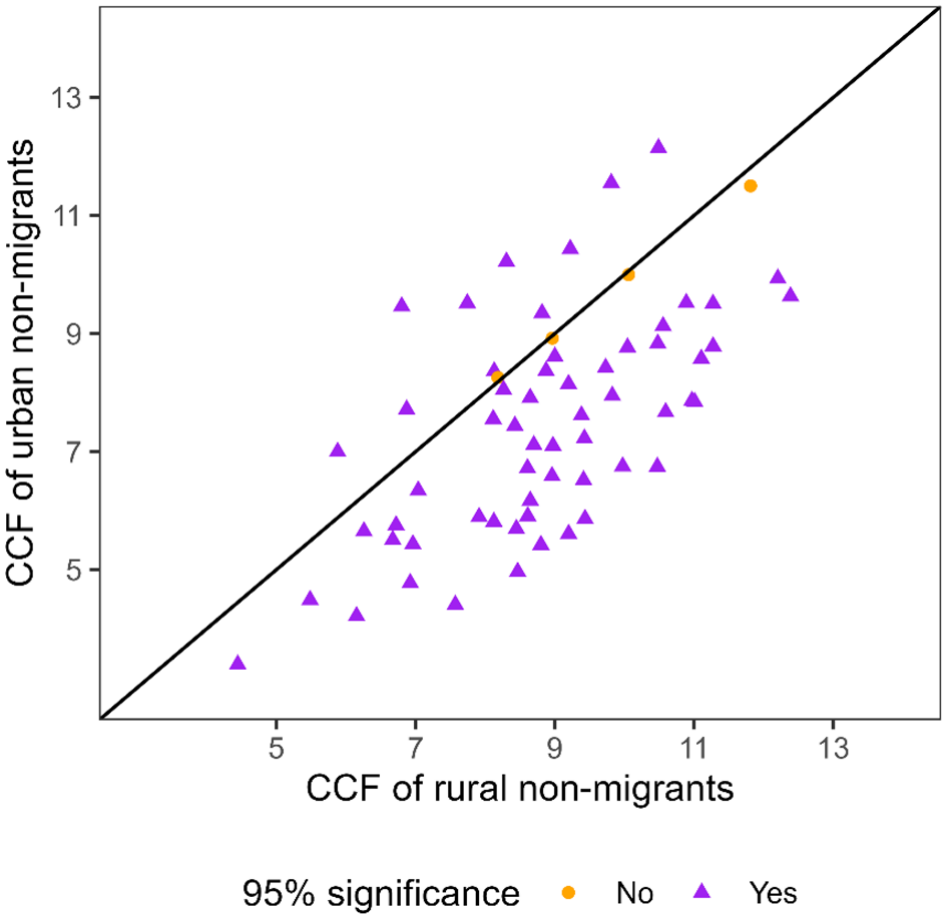
Rural and urban non-migrant male fertility, based on completed fertility of 50–64-year-old men determined at men’s current place of residence

Focusing on non-migrants in rural and urban populations, we find that rural male fertility (measured at time of survey) is higher than urban across countries (and years), except for in ten surveys (Fig. 3). These findings are corroborated by examining rural/urban TFR (see Appendix Fig. 7). This broadly suggests that the differences between rural and urban male fertility are independent of internal migration, as noted in developed countries (Kulu, 2013). Essentially, non-migrant male fertility accounts for the fertility levels in each rural/urban area (Appendix Fig. 8), which is not surprising considering the high proportion of non-migrants in our sample (see Table 1).

### Internal Migrants Have Lower Fertility than Rural Non-migrants

**Fig. 4:**
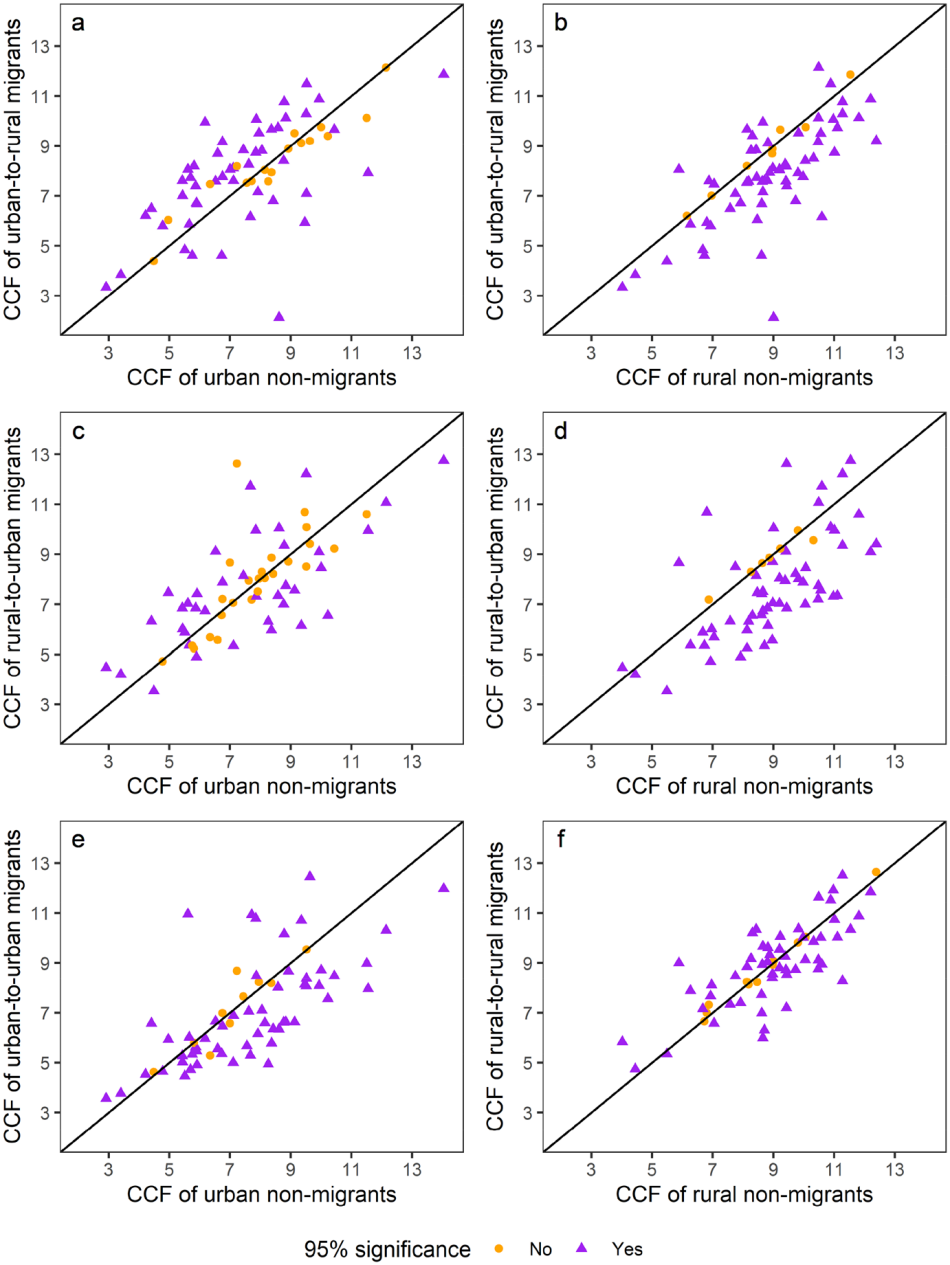
Male migrant completed fertility levels determined at men’s current place of residence, in comparison with completed fertility in their places of origin and destination

Male migrants have different CCF levels as compared to non-migrants in both rural and urban areas. In comparison with rural non-migrants (Fig. 4b, d), male migrants between the urban and rural sectors have lower fertility, capturing selection (of lower fertility among rural-to-urban migrants) and possibly disruptive mechanisms (among urban-to-rural migrants). In other words, regardless of direction of migration flow, migrants have lower fertility as compared to rural non-migrants. This suggests that neither socialisation of migrants from the rural sector, nor adaptation of migrants to their rural environment are at play. In contrast to Fig. 4b, urban-to-rural male migrants have higher fertility than urban non-migrants (Fig. 4a), indicating that they are a select group, or that there is some adaptation to higher rural fertility, even if it is not as high as rural non-migrant levels. For instance, urban-to-rural migrants are less educated than urban non-migrants, but more educated than rural non-migrants (see Appendix Table 4). Also, the proportion of urban-to-rural male migrants in union who are polygamous is more similar to that of rural non-migrant men than to that of urban non-migrant men.

When we compare migrant CCF to completed fertility of non-migrants at their destination (Fig. 4c, d), we note that urban-to-rural men have lower fertility than rural non-migrant men, but rural-to-urban migrants do not show a similarly clear pattern. In contrast, rural-to-urban migrant men tend to have higher CCF than non-migrant urban men. Migrants moving within the urban sector have lower fertility than non-migrant urban men (Fig. 4e), suggesting selection or disruption mechanisms are at play. Considering intra-urban migrants do not change their environments, there is little adaptation involved. Moreover, they are already socialised in urban settings, although we note that there may be some changes if the migration is to or from the capital city or a very large city, which tend to have different environments than small towns. Intra-urban migrants may be different to non-migrant urbanites, and their migration experience may lead them to postpone fathering. For instance, urban-to-urban migrants are better educated and less polygamous than urban non-migrants (see Appendix Table 4). In contrast, migrants moving within the rural sector seem to have similar CCF to rural non-migrants (Fig. 4f), possibly reflecting strong rural socialisation. This implies that high rates of migration within the rural sector would not affect rural fertility levels, while high rates of intra-urban migration would contribute to lower urban fertility. These patterns of fertility by migration status remain even when the age range is extended to include younger men from age 45 (Appendix Fig. 9), although the values of the rates are lower when younger men are included in the sample (since younger men are less likely to have completed their fertility. Similarly, when we consider only surveys with at least 30 men in each category of migration status, the results remain similar, although the number of surveys decreases dramatically in the rural-to-urban category (see Appendix Fig. 10).

These results are also largely corroborated by examination of TFR. The only contradictory evidence we note is that urban-to-urban migration, based on TFR (a period measure), seems to be higher than that of non-migrants within the urban sector. However, using our cohort measure of fertility, CCF, with the same subsample of surveys, urban-to-urban migrant fertility seems to be lower than non-migrant urban fertility (Appendix Fig. 11).

### Largest Differences in Migrant Fertility are Among Men with Secondary or Higher Education

Turning to the models, estimates in Table 2 suggest that the number of children ever born to 50–64-year-old men in the urban sector is lower than in rural SSA, consistent with the results presented in Figs. 1, 2 and 3. On average, urban men have 1.8 [95% CI 1.7–1.9] children less than rural men during their reproductive lives (Model 0 in Table 2). Model 1 in Table 2 indicates that non-migrant urban men have on average 1.7 [95% CI 1.6–1.9] children less than non-migrant rural men. Rural-to-urban migrant men have 1.4 [95% CI 1.1–1.6] less children and urban-to-rural 1.2 [95% CI 1.1–1.4] children less than non-migrant rural men, on average. This suggests that urban-to-rural migrants are more similar to their rural non-migrant counterparts than to urban non-migrant men. These findings reflect what we see in Fig. 4: rural-to-urban migrant men have lower CCF than rural non-migrants, yet higher than urban non-migrants. Similarly, urban-to-rural migrant men have lower CCF than rural non-migrants but higher than urban non-migrant. Migrants within the rural sector have almost the same number of children as rural non-migrants (0.2 less [95% CI 0.1–0.4]), while migrants within the urban sector are the most different to rural non-migrants, having on average 2.6 [95% CI 2.4–2.7] children less.

**Table 2:** Model estimates of the effect of migration on completed fertility of men aged 50–64. Dependent variable: children ever born

	Model 0		Model 1		Model 2		Model 3	
	*β* (std. err.)	95% C.I	*β* (std. err.)	95% C.I	*β* (std. err.)	95% C.I	*β* (std. err.)	95% C.I
Intercept	4.802	4.0–5.6	4.704	3.9–5.5	4.609	3.8–5.4	4.614	3.8–5.4
	(0.396)		(0.394)		(0.391)		(0.392)	
*Place of residence*								
Rural (reference)								
Urban	− 1.786	− 1.9 to − 1.7						
	(0.057)							
*Migration status*								
Rural non-migrant (reference)								
Urban non-migrant			− 1.724	− 1.9 to − 1.6	− 1.332	− 1.5 to − 1.2	− 0.903	− 1.2 to − 0.6
			(0.085)		(0.087)		(0.152)	
Urban to rural			− 1.238	− 1.4 to − 1.1	− 0.955	− 1.1 to − 0.8	− 0.874	− 1.1 to − 0.5
			(0.088)		(0.088)		(0.152)	
Rural to urban			− 1.369	− 1.6 to − 1.1	− 1.065	− 1.3 to − 0.8	− 0.902	− 1.4 to − 0.4
			(0.123)		(0.123)		(0.231)	
Urban to urban			− 2.565	− 2.7 to − 2.4	− 1.923	− 2.1 to − 1.8	− 1.692	− 2.1 to − 1.3
			(0.081)		(0.086)		(0.211)	
Rural to rural			− 0.230	− 0.4 to − 0.1	− 0.176	− 0.3 to − 0.0	− 0.597	− 0.9 to − 0.3
			(0.085)		(0.084)		(0.138)	
*Education level*								
No education (reference)								
Primary					− 0.547	− 0.7 to − 0.4	− 0.542	− 0.7 to − 0.4
					(0.069)		(0.090)	
Secondary+					− 1.536	− 1.7 to − 1.4	− 1.437	− 1.7 to − 1.2
					(0.077)		(0.122)	
*Interaction migration and education*								
Urban non-migrant × Primary							− 0.353	− 0.7 to 0.1
							(0.217)	
Urban-to-rural × Primary							− 0.292	− 0.7 to 0.1
							(0.212)	
Rural-to-urban × Primary							− 0.286	− 0.9 to 0.3
							(0.307)	
Urban-to-urban × Primary							− 0.122	− 0.6 to 0.4
							(0.261)	
Rural-to-rural × Primary							0.561	0.2–0.9
							(0.183)	
Urban non-migrant × Secondary+							− 0.797	− 1.2 to − 0.4
							(0.215)	
Urban-to-rural × Secondary+							− 0.014	− 0.5 to 0.4
							(0.223)	
Rural-to-urban × Secondary+							− 0.216	− 0.8 to 0.4
							(0.314)	
Urban-to-urban × Secondary+							− 0.388	− 0.9 to 0.1
							(0.250)	
Rural-to-rural × Secondary+							0.769	0.3–1.2
							(0.236)	
*Number of previous partners*								
Mothers	2.302	2.2–2.4	2.310	2.2–2.4	2.308	2.2–2.4	2.307	2.2–2.4
	(0.032)		(0.032)		(0.069)		(0.031)	
AIC	133,309.00		133,619.02		133,211.01		133,186.30	
BIC	134,256.40		133,998.73		133,606.87		133,662.95	
Log Likelihood	− 66,911.50		− 66,762.51		− 66,556.50		− 66,534.15	
Deviance	382,924.04		378,166.51		371,685.69		370,989.15	
Num. obs	23,835		23,835		23,835		23,835	
Fixed effects								
Country	Yes		Yes		Yes		Yes	
Survey year	Yes		Yes		Yes		Yes	

Numbers in parenthesis are standard errors

When we further account for education level of the men, this relationship between fertility and migration is maintained though coefficients are slightly lower. Estimates from Model 2 (in Table 2) show that men with at least secondary education have on average 1.5 [95% CI 1.4–1.7] children less than those with no education and 0.5 children less than those with primary education. In Model 2, and across all models, we find, as expected, that the number of women with whom a man has partnered increases the number of children fathered. On average, each man has 2.3 [95% CI 2.2–2.4] children with each additional woman he has fathered.

**Fig. 5:**
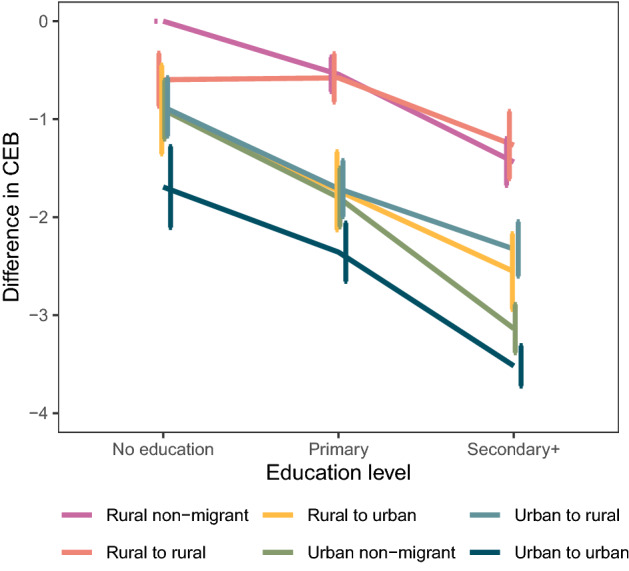
Effect of the interaction of education and migration on the number of children ever born (CEB) to men aged 50–64 (Model 3 in Table 2). *Note*: Reference category is rural non-migrants with no education

Since the probability of migration is often related to education in an inverse-U shape (Dustmann & Glitz, 2011), and the relationship between fertility and education further differs by rural/urban area (Shapiro & Tenikue, 2017), we include interaction terms of migration and education in Model 3 (Table 2). To facilitate correct interpretation of the coefficients, we present in Fig. 5 the interaction effect of education and migration status. The number of children ever born (CEB) decreases by level of education, where the most educated men have lower fertility levels across all migration statuses. Rural non-migrants and rural-to-rural migrants have clearly higher fertility than all other migrant statuses with primary and secondary education. Among men with no education, differences between migration statuses are small. The largest difference is between non-migrant rural dwellers and urban-to-urban migrants. In contrast, the differences are large among men with at least secondary education, with urban-to-urban migrants having the lowest number of CEB. We observe that rural non-migrants with no education have on average between 0.7 and one more child than men with no education of other migration statuses, and the difference increases when comparing men with primary education. Among men with at least secondary education, on average urban-to-rural and rural-to-urban migrants have two less children, and urban-to-urban migrants and urban non-migrants at least three fewer children, compared to rural non-migrants. There are no differences in fertility among between-sector migrant men with primary or secondary education. These results are consistent even when we expand the sample size by including men from age 45 too (see Appendix Table 5 with a robustness check of Model 3).

### Rural-To-Urban Migrants are a Select Group

We next consider the timing of migration in comparison with the timing of the birth of the youngest child to assess what mechanisms may be driving a differential migration-fertility relationship. It should be noted that the number of observations in these models (Table 3) decreases as not all DHS include information on the date of birth of the last child, and childless men are also excluded. We note that since the level of childlessness in our sample is low, it is very unlikely that our results are biased. In Model 4, we disaggregate the migration flows according to whether a man moved before or after the birth of his last child. All the coefficients of migration after the last birth are lower than before the last birth, indicating that migrants are a select group. We find that in comparison with rural non-migrants, migrant men who moved between sectors after the birth of their last child (mostly completed their fertility) had on average 2.6 fewer children, suggesting a selection effect. When we consider migrants who move before the birth of their last child, their fertility is lower than rural non-migrants, indicating modest adaptation. Lastly, men who migrated within the rural sector before the last child birth have more children than their rural non-migrant counterparts (0.5 [95% CI 0.2–0.7]), suggesting perhaps that they are not affected by disruption. Model 5 in Table 3 controls for education,^4^ and overall, coefficients decrease slightly, but the relations remain. The selection effects we find are in line with previous findings in Burkina Faso, where both men and women migrants were found to have lower fertility as compared to their place of origin (Pongi Nyuba, 2019). In Model 6 in Table 3, we add ranges of number of years since migration to assess whether men adapt to their destination. Coefficients are comparable between Model 5 and Model 6, but slightly lower in the latter, and the Akaike information criterion (AIC) and Bayesian information criteria (BIC) are very similar. The coefficients associated with duration since migration show that more recent migrants have higher fertility than men who migrated more than five years ago suggesting little disruption and some adaptation.

**Table 3:** Effect of time of migration on completed fertility of men aged 50–64 according to flux of migration. Dependent variable: children ever born

	Model 4		Model 5		Model 6	
	*β* (std. err.)	95% C.I	*β* (std. err.)	95% C.I	*β* (std. err.)	95% C.I
Intercept	7.912	7.6–8.2	8.285	7.9–8.6	8.291	7.9–8.6
	(0.155)		(0.156)		(0.156)	
*Rural non-migrant (reference)*						
Urban non-migrant	− 1.869	− 2.1 to − 1.7	− 1.458	− 1.6 to − 1.3	− 1.455	− 1.6 to − 1.3
	(0.098)		(0.099)		(0.099)	
After last birth Urban-to-rural	− 2.573	− 2.9 to − 2.2	− 2.189	− 2.5 to − 1.9	− 1.990	− 2.4 to − 1.6
	(0.170)		(0.169)		(0.220)	
After last birth Rural-to-urban	− 2.582	− 3.2 to − 2.0	− 2.222	− 2.8 to − 1.7	− 2.033	− 2.7 to − 1.4
	(0.291)		(0.289)		(0.318)	
After last birth Urban-to-urban	− 3.104	− 3.4 to − 2.8	− 2.403	− 2.7 to − 2.1	− 2.203	− 2.6 to − 1.8
	(0.141)		(0.145)		(0.201)	
After last birth Rural-to-rural	− 1.520	− 1.9 to − 1.2	− 1.397	− 1.7 to − 1.1	− 1.213	− 1.7 to − 0.8
	(0.178)		(0.176)		(0.224)	
Before last birth Urban-to-rural	− 0.692	− 0.9 to − 0.5	− 0.423	− 0.6 to − 0.2	− 0.156	− 0.5 to 0.2
	(0.115)		(0.115)		(0.177)	
Before last birth Rural-to-urban	− 0.711	− 1.1 to − 0.4	− 0.449	− 0.8 to − 0.1	− 0.185	− 0.6 to 0.3
	(0.180)		(0.179)		(0.224)	
Before last birth Urban-to-urban	− 2.379	− 2.6 to − 2.2	− 1.749	− 1.9 to − 1.5	− 1.477	− 1.8 to − 1.1
	(0.111)		(0.115)		(0.178)	
Before last birth Rural-to-rural	0.456	0.2–0.7	0.499	0.3–0.7	0.770	0.4–1.1
	(0.115)		(0.114)		(0.177)	
*No education (reference)*						
Primary			− 0.751	− 0.9 to − 0.6	− 0.750	− 0.9 to − 0.6
			(0.082)		(0.082)	
Secondary+			− 1.687	− 1.9 to − 1.5	− 1.681	− 1.9 to − 1.5
			(0.088)		(0.082)	
Number of mothers	2.069	1.9–2.2	2.076	1.9–2.2	2.076	2.0–2.2
	(0.042)		(0.041)		(0.041)	
0 years since migration (reference)						
1 to 5 years					− 0.078	− 0.4 to 0.3
					(0.187)	
6 to 10 years					− 0.395	− 0.8 to − 0.0
					(0.185)	
11 to 15 years					− 0.442	− 0.8 to − 0.1
					(0.192)	
16 to 20 years					− 0.518	− 0.9 to − 0.1
					(0.197)	
More than 20 years					− 0.130	− 0.4 to 0.2
					(0.143)	
AIC	98,405.58		98,039.86		98,035.06	
BIC	98,700.93		98,350.75		98,384.81	
Log Likelihood	− 49,164.79		− 48,979.93		− 48,972.53	
Deviance	279,394.96		273,567.35		273,336.52	
Num. obs	17,540		17,540		17,540	
Fixed effects						
Country	Yes		Yes		Yes	
Survey year	Yes		Yes		Yes	

Numbers in parenthesis are standard errors

## Conclusion

Male fertility remains a little explored field, and sub-national differences in SSA have not previously been analysed. We find that male cohort fertility has declined, particularly in urban areas. Broadly across SSA, the decline in male fertility is slower in the rural sector than in the urban sector. Rural CCF remains stable over the period examined, at between eight to nine children. Even when we exclude internal migrants, rural CCF is higher than urban CCF, suggesting that migration between the areas is not influencing these trends, in line with recent findings on the low contribution of internal migration to urbanisation (Menashe-Oren & Bocquier, 2021). Certainly, if migrants were a larger proportion of the population whether in rural/urban areas, the effect of their movements could be felt.

In a first attempt at examining the relationship between migration and male fertility in SSA, we find that between- sector migrant men have lower fertility than their rural non-migrant counterparts but tend to be higher than urban non-migrants. In contrast, rural-to-rural migrant men have similarly high fertility as non-migrant rural men and urban-to-urban migrants have lower fertility than urban non-migrants. Fertility among between- sector migrants is most different to rural fertility levels notwithstanding the direction of migration flow. This suggests that rural-to-urban migrants are a select group, and that urban-to-rural migrants do not adapt much to their rural destination. It is possible that urban-to-rural migrant men are actually return migrants who maintained their rural preferences as they were socialised in the rural sector but adapted to some extent to urban fertility levels. We corroborate this in our final analysis examining the timing of migration in relation to fathering. Although we do not have birth histories for the men included in our analysis, we are able to identify the time of the last birth in 61% of our sample. Of those for which we have the timing of last birth, 16% are migrants. Around 71% of migrant men migrated before the birth of their last child. Men who migrated after the birth of their last child have on average about 1.8 children less than men who migrated before the birth of their last child (Appendix Table 6). This indicates that most men migrate at an earlier stage of their reproductive lives.

Once we account for education level, we find that more educated men have fewer children, while also controlling for the number of partners with whom they had fathered children. This is in contrast to evidence from Europe of a positive relationship between fertility and education among men as compared to a negative one found amongst women (Kravdal & Rindfuss, 2008). The education level of migrants is particularly important when comparing migrant fertility to fertility levels in the urban sector. Migrant men who have at least secondary education have significantly higher CCF than urban non-migrants and urban-to-urban migrants. In contrast, fertility levels are less divergent among men with lower levels of education. This suggests that within-sector migrant men adapt quicker to fertility at destination when they have lower levels of education, or that when with higher education they are a select group, with higher fertility desires. Socialisation may also be at play, with low educated migrants maintaining the fertility levels of place of origin.

We find that the mechanisms of selection and disruption play key roles in migrant men’s fertility, while adaptation and socialisation are less prevalent. The results suggest that selection is central in lowering rural-to-urban and urban-to-urban migrant fertility. In other words, the characteristics associated with fertility levels (whether directly related, such as age at marriage, or indirectly related, such as ease of dealing with change) of these internal migrants, even before they move, are generally different to non-migrant men’s characteristics. Intra-urban migrants have higher educational attainment than urban non-migrants as do rural-to-urban migrants compared to rural non-migrants (see Appendix Table 4). As mentioned above, men with higher educational levels tend to have lower fertility rates than men with lower educational levels. Also, urban-to-urban migrants and rural-to-urban migrants are less polygamous than urban non-migrants and, on average, their fertility was completed earlier with their last child being born earlier than among urban non-migrants. It is possible that selection is at play also with urban-to-rural migration flows, though these men have higher fertility than urban non-migrants. We posit that urban-to-rural migrants are a distinct group of men who are likely return migrants—with select characteristics (associated with lower fertility) in comparison with rural non-migrants at the time of their initial move. The lack of selection effects (and disruption) in rural-to-rural migration is striking: fertility preferences are similar among migrants and rural non-migrants (whether at origin or destination). Rural fertility norms appear dominant, and less flexible than urban fertility values.

Further research including more precise birth records of men are needed to evaluate the mechanisms behind the relationship between male fertility and internal migration more thoroughly. In particular, precise information about the timing and order of migration events in relation to birth dates would be beneficial. It is possible that these mechanisms may also differ according to country context. Moreover, a limitation to our analysis is that we were not able to account for a full migration history, but only the most recent move: some migrants moved multiple times or were circular migrants. Their fertility may also be different, reflecting “double selection”, diffusion, or “re-adaptation” (Pongi Nyuba, 2019), as appears to be the case in our results with urban-to-rural migrants.

Our analysis focused on movement between rural and urban sectors. Nonetheless, it is possible that fertility differs also between semi-urban or semi-rural sectors, over a gradient of urbanicity. While the DHS includes four categories of previous residence, we were limited to collapsing the categories due to small sample sizes (see Table 1). The fertility of a rural male migrant may differ if his destination is a small town as compared to the capital city. We contend that considering women in SSA living in towns and small cities have similar age-specific fertility rates as in major cities (Stecklov & Menashe-Oren, 2019), our analysis likely captures the core of the fertility-migration relationship.

A final limitation in our analysis derives from using CCF which reflects distant fertility levels, although it does capture smoother trends rather than short-term variation. Our robustness test using TFR for men suggests that the differences in rural and urban male fertility we find are also relevant today.

We have contributed to an understanding of within-country male fertility in SSA and taken a first broad look at the relationship between internal migration and fertility among men. Men play a key role in fertility decisions, and even have higher fertility rates than women (Schoumaker, 2019), and are committed to their families as much as women (Bankole & Singh, 1998; Forste, 2002; Ratcliffe et al., 2001). Their variation in fertility needs to be accounted for.

## Figures and Tables

**Fig. 6:**
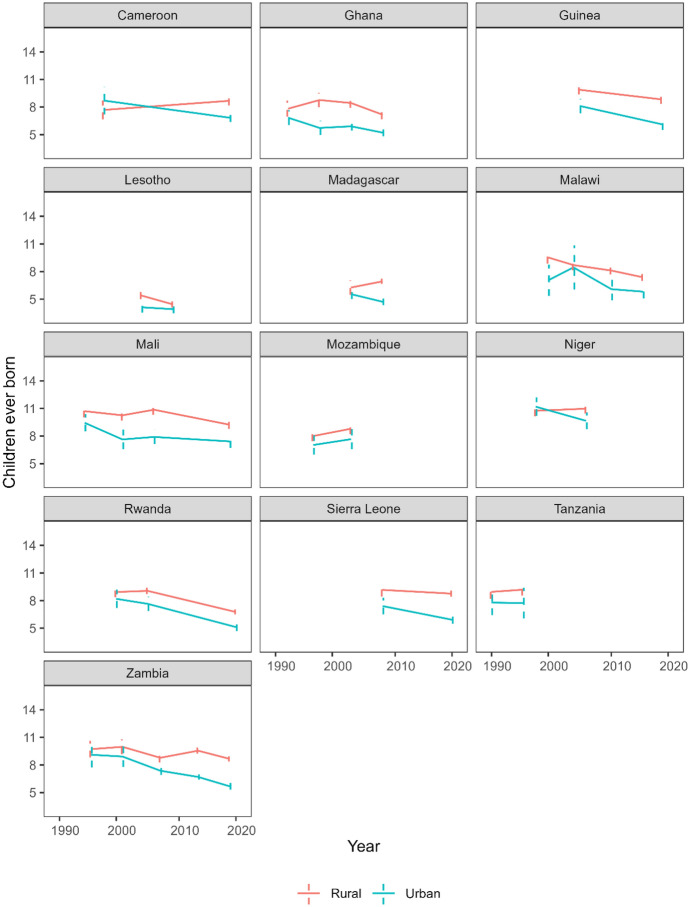
Rural and urban male fertility trends over time in countries with at least two DHS, based on completed fertility of 50–64-year-old men. 95% confidence intervals

**Fig. 7:**
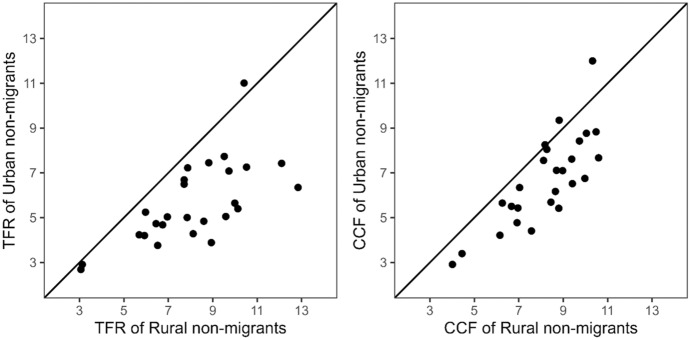
Comparison of non-migrant fertility in rural and urban sectors using two different measures of male fertility: TFR and CCF. *Note*: CCF estimates plotted here only include the surveys for which we could also estimate TFR

**Fig. 8:**
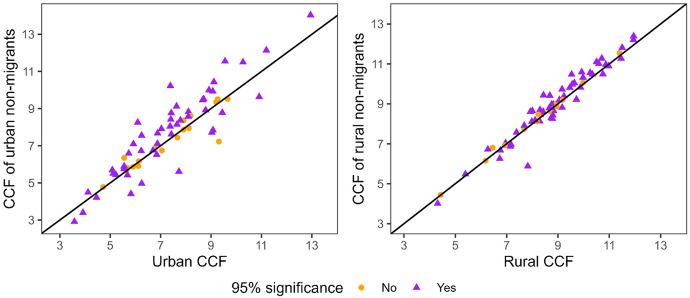
Comparison of non-migrant and total male fertility in urban and rural areas

**Fig. 9:**
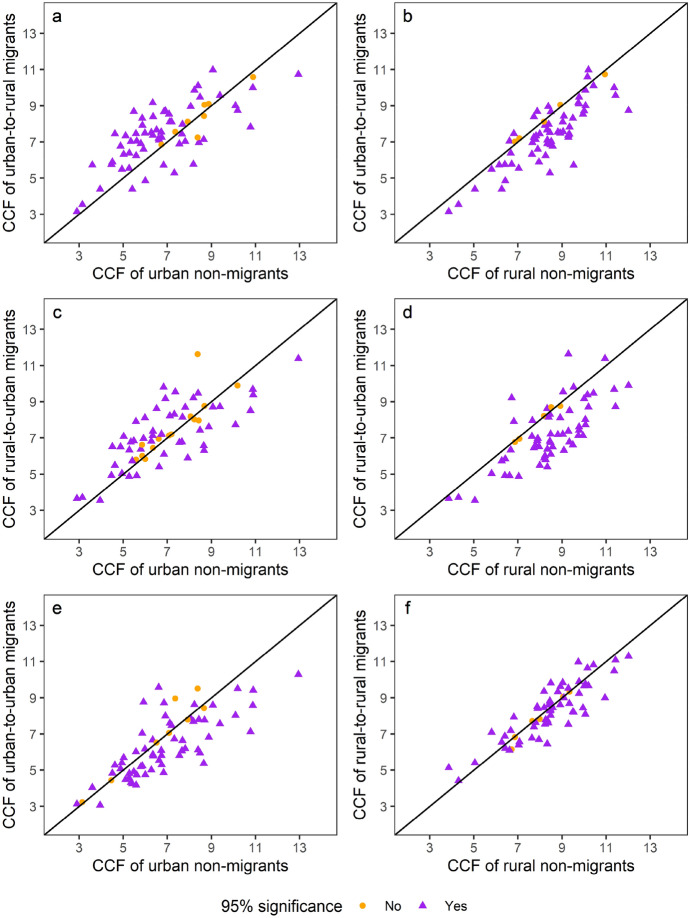
Male migrant completed fertility levels in comparison to completed fertility in their places of origin and destination, using wider age group (45–64)

**Fig. 10:**
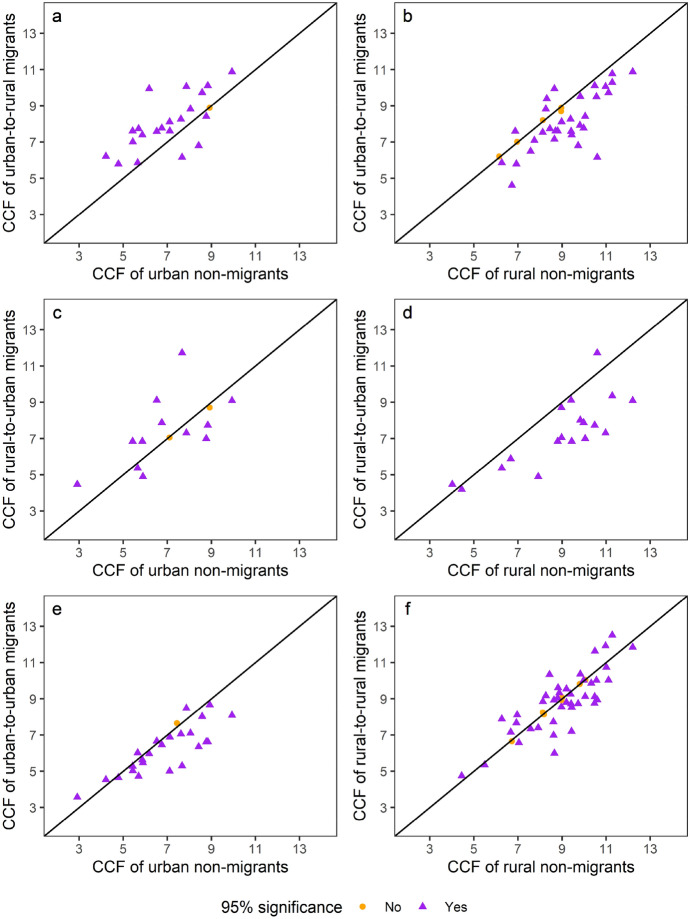
Male migrant completed fertility levels in comparison to completed fertility in their places of origin and destination, limited to samples with at least 30 men

**Fig. 11:**
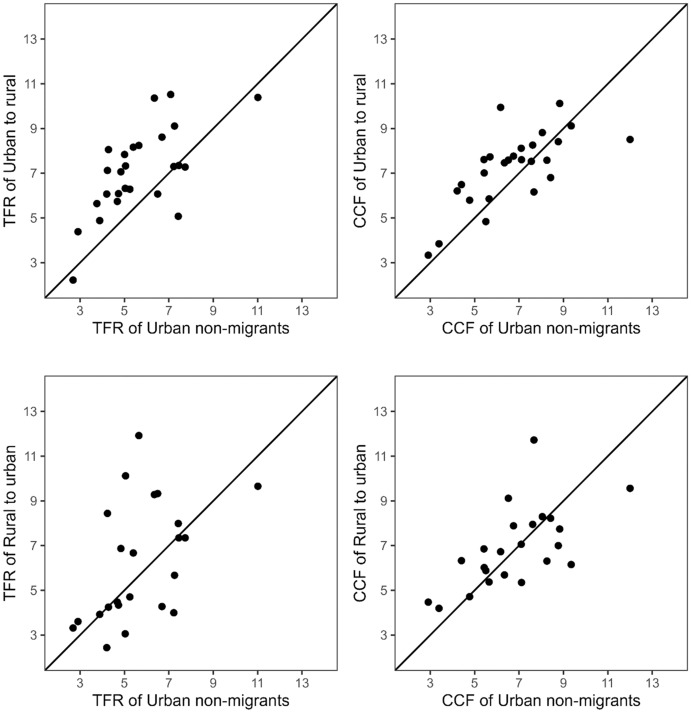

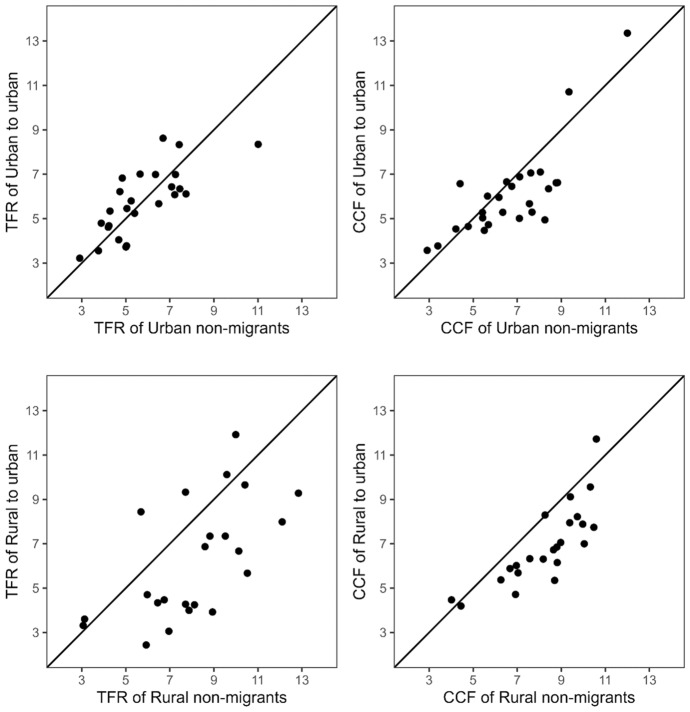

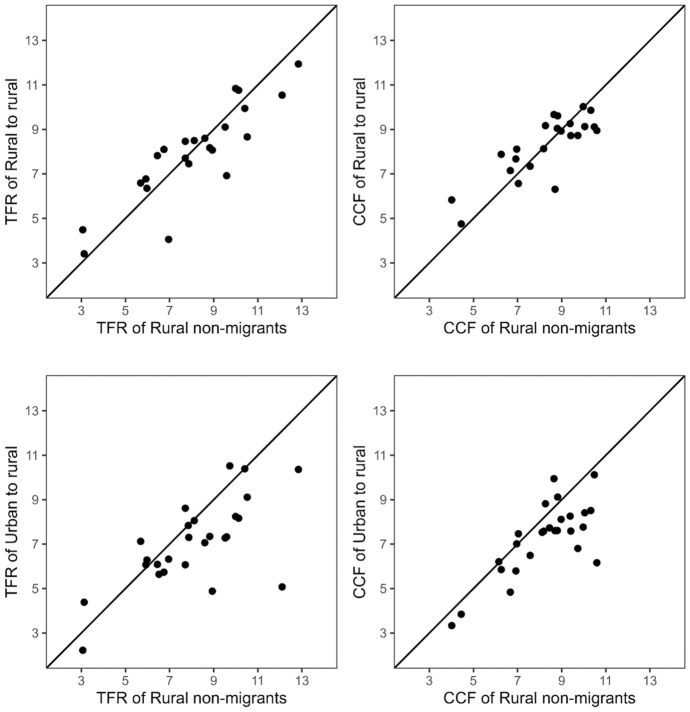
Comparison of fertility by migration status using two different measures of male fertility: TFR and CCF

## Appendix

See Figs. 6, 7, 8, 9, 10, 11 and Tables 4, 5, 6.

**Table 4:** Selected descriptive statistics by migration status

	Non-migrants		Migrants			
			Between areas		Within areas	
	Urban	Rural	To urban	To rural	To urban	To rural
*Percentages*						
Marital status						
In union	89.9	93.2	90.6	91.5	90.9	92.4
Polygyny	19.7	25.4	18.5	22.4	13.0	21.3
Fertility						
Childlessness	2.3	2.1	2.4	1.6	1.6	1.8
Education						
No education	27.9	54.0	29.4	33.6	12.2	37.5
Primary	29.0	34.7	35.9	33.6	24.6	45.8
Secondary and higher	43.4	11.4	34.7	32.8	63.2	16.7
*Averages*						
Fertility						
Age at first birth	27.5	26.4	27.1	26.9	27.7	26.1
Age at last birth	44.7	45.9	43.9	44.8	43.2	45.4
CCF	7.5	9.0	7.4	8.0	6.4	8.9
Migration						
Years since last migration			19.0	15.6	15.4	16.0

These descriptive statistics refer to the time of the survey

**Table 5:** Model 3 estimates of the effect of migration on completed fertility of men. Dependent variable: children ever born

	50–64 age group		45–64 age group	
	*β* (std. err.)	95% C.I	*β* (std. err.)	95% C.I
Intercept	4.614	3.8 to 5.4	4.945	4.4–5.5
	(0.392)		(0.284)	
*Migration status*				
Rural non-migrant (reference)				
Urban non-migrant	− 0.903	− 1.2 to − 0.6	− 0.821	− 1.0 to − 0.6
	(0.152)		(0.112)	
Urban to rural	− 0.874	− 1.1 to − 0.5	− 0.809	− 1.0 to − 0.6
	(0.152)		(0.112)	
Rural to urban	− 0.902	− 1.4 to − 0.4	− 0.992	− 1.3 to − 0.7
	(0.231)		(0.172)	
Urban to urban	− 1.692	− 2.1 to − 1.3	− 1.637	− 1.9 to − 1.3
	(0.211)		(0.152)	
Rural to rural	− 0.597	− 0.9 to − 0.3	− 0.517	− 0.7 to − 0.3
	(0.138)		(0.100)	
*Education level*				
No education (reference)				
Primary	− 0.542	− 0.7 to − 0.4	− 0.517	− 0.6 to − 0.4
	(0.090)		(0.064)	
Secondary+	− 1.437	− 1.7 to − 1.2	− 1.446	− 1.6 to − 1.3
	(0.122)		(0.083)	
*Interaction migration and education*				
Urban non-migrant × Primary	− 0.353	− 0.7 to 0.1	− 0.470	− 0.7 to − 0.2
	(0.217)		(0.154)	
Urban-to-rural × Primary	− 0.292	− 0.7 to 0.1	− 0.378	− 0.7 to − 0.2
	(0.212)		(0.153)	
Rural-to-urban × Primary	− 0.286	− 0.9 to 0.3	− 0.309	− 0.7 to 0.1
	(0.307)		(0.222)	
Urban-to-urban × Primary	− 0.122	− 0.6 to 0.4	− 0.174	− 0.5 to 0.2
	(0.261)		(0.188)	
Rural-to-rural × Primary	0.561	0.2–0.9	0.383	0.1–0.6
	(0.183)		(0.130)	
Urban non-migrant × Secondary+	− 0.797	− 1.2 to − 0.4	− 0.700	− 1.0 to − 0.4
	(0.215)		(0.151)	
Urban-to-rural × Secondary+	− 0.014	− 0.5 to 0.4	0.067	− 0.2 to 0.4
	(0.223)		(0.157)	
Rural-to-urban × Secondary+	− 0.216	− 0.8 to 0.4	− 0.026	− 0.5 to 0.4
	(0.314)		(0.225)	
Urban-to-urban × Secondary+	− 0.388	− 0.9 to 0.1	− 0.345	− 0.7 to 0.0
	(0.250)		(0.177)	
Rural-to-rural × Secondary+	0.769	0.3–1.2	0.582	0.3–0.9
	(0.236)		(0.162)	
*Number of previous partners*				
Mothers	2.307	2.2–2.4	2.245	2.2–2.3
	(0.031)		(0.023)	
AIC	133,186.30		234,031.08	
BIC	133,662.95		234,542.27	
Log Likelihood	− 66,534.15		− 116,956.54	
Deviance	370,989.15		592,925.07	
Num. obs	23,835		42,789	
Fixed effects				
Country	Yes		Yes	
Survey year	Yes		Yes

**Table 6:** Effect of the timing of migration in relation to last birth on completed fertility of migrant men aged 50–64. Dependent variable: children ever born

	Model A1		Model A2	
	*β* (std. err.)	95% C.I	*β* (std. err.)	95% C.I
Intercept	7.446	6.9 to 8.0	6.239	6.0–6.5
	(0.272)		(0.109)	
*Urban to rural (reference)*				
Rural to urban	− 0.260	− 0.6 to 0.1	− 0.422	− 0.7 to − 0.1
	(0.168)		(0.166)	
Urban to urban	− 1.517	− 1.7 to − 1.3	− 1.517	− 1.7 to − 1.3
	(0.113)		(0.115)	
Rural to rural	0.984	0.7 to 1.2	1.047	0.8–1.3
	(0.127)		(0.119)	
*After (reference)*				
Before	1.814	1.6 to 2.0	1.845	1.7–2.0
	(0.096)		(0.098)	
AIC	40,878.94		41,326.08	
BIC	41,100.27		41,367.58	
Log Likelihood	− 20,407.47		− 20,657.04	
Deviance	104,151.74		111,363.88	
Num. obs	7455		7455	
Fixed effects				
Country	Yes		No	
Survey year	Yes		No	

Numbers in parenthesis are standard errors

## Footnotes

1 Three other methods have been proposed to estimate total fertility rates (TFR) for males (Schoumaker, 2017). The first method is the own-child method, based on a list of all children in the household, their ages and whether their father is in the household. With this method, the record of children is limited to those living with the father in his current household and we do not know of any children he may have had previously, before or after migration, or out of the household. The second method is the crisscross method, based on the number of children ever born among men in two successive surveys spaced around five years apart. This method poses restrictions on the number and timing of surveys which would significantly reduce our sample size. We cannot use either of these methods because it is not possible to link fertility to information on migration. The third method is based on the date of birth of the last (youngest) child computed as the ratio of the number of last births to the exposure in an age group in a period. We use this method to test for robustness of our results. However, because of its limitations we do not use this method in our main analysis. A first limitation is in the number of surveys which include the date of birth of the child (reducing sample size). Second, this method underestimates fertility rates, since it is not based on birth histories, and this is especially apparent in high-fertility countries such as those we examine. A third limitation is that we cannot examine trends with this method, since the DHS began to collect the information needed to estimate the TFR only in recent years.

2 This estimate of childlessness is likely biased since, on the one hand, childlessness is often under-reported in regions where it is stigmatised, while on the other hand, children who have died are often omitted, and men may also not be aware of children they have fathered.

3 For example, 2010 CCF is approximately comparable to male TFR between 1995 and 2000, when the 50–64-year-old men were between ages 35 and 50. We are unable to compare TFR for the same period, since the DHS surveys we could use to estimate TFR are only from 2005 on.

4 We do not include interactions between education and migration status in this model since this would lead to very few cases in some combined categories.

## References

[CR1] Abel GJ, Sander N (2014). Quantifying global international migration flows. Science.

[CR2] Adepoju A (1998). Linkages between internal and international migration: The African situation. International Social Science Journal.

[CR3] Agadjanian V, Ezeh AC (2000). Polygyny, gender relations, and reproduction in Ghana. Journal of Comparative Family Studies.

[CR4] Agadjanian V, Yabiku ST, Cau B (2011). Men’s migration and women’s fertility in rural mozambique. Demography.

[CR5] Bach RL (1981). Migration and fertility in Malaysia: A tale of two hypotheses. The International Migration Review.

[CR6] Bankole A, Singh S (1998). Couples’ fertility and contraceptive decision-making in developing countries: hearing the man’s voice. International Family Planning Perspectives.

[CR7] Basu, B. (1999). Relationship between migration and fertility decisions in rural sectors of LDC’s. *Journal of Economic Development, 24*(1), 77–96.

[CR8] Beauchemin C (2011). Rural–urban migration in West Africa: Migration trends and economic situation in Burkina Faso and Cote d’Ivoire. Population, Space and Place.

[CR9] Beauchemin C, Bocquier P (2004). Migration and urbanisation in francophone West Africa: An overview of the recent empirical evidence. Urban Studies.

[CR10] Beguy D, Bocquier P, Zulu EM (2010). Circular migration patterns and determinants in Nairobi slum settlements. Demographic Research.

[CR11] Beine M, Docquier F, Schiff M (2013). International migration, transfer of norms and home country fertility. Canadian Journal of Economics.

[CR12] Bernard A, Bell M, Charles-edwards E (2014). Life-course transitions and the age profile of internal migration. Population and Development Review.

[CR14] Bertoli S, Marchetta F (2015). Bringing It all back home—Return migration and fertility choices. World Development.

[CR15] Bocquier, P., Menashe-Oren, A., & Nie, W. (2023). Migration’s contribution to the urban transition: Direct census estimates from Africa and Asia. *Demographic Research* (forthcoming).

[CR16] Brockerhoff M, Bilsborrow RE (1996). Migration and the fertility transition in African Cities. Migration, urbanisation and development: New directions and issues.

[CR17] Brockerhoff M, Eu H (1993). Demographic and socioeconomic determinants of female rural to urban migration in Sub- Saharan Africa. International Migration Review.

[CR18] Cantalini S, Panichella N (2019). The fertility of male immigrants: A comparative study on six Western European countries. European Societies.

[CR19] Chattopadhyay A, White MJ, Debpuur C (2006). Migrant fertility in Ghana: Selection versus adaptation and disruption as causal mechanisms. Population Studies.

[CR20] Clark SJ, Collinson MA, Kahn K, Drullinger K, Tollman SM (2007). Returning home to die: Circular labour migration and mortality in South Africa. Scandinavian Journal of Public Health.

[CR21] Corker J (2017). Fertility and child mortality in Urban West Africa: Leveraging geo-referenced data to move beyond the urban/rural dichotomy. Population, Space and Place.

[CR22] Coulter R, Scott J (2015). What motivates residential mobility? Re-examining self-reported reasons for desiring and making residential moves. Population, Space and Place.

[CR23] Deshingkar, P., & Grimm, S. (2005). *Internal migration and development: A global perspective* (Vol. 19).

[CR24] Doepke M, Tertilt M (2018). Women’s empowerment, the gender gap in desired fertility, and fertility outcomes in developing countries. AEA Papers and Proceedings.

[CR25] Duncan RP, Perrucci CC (1976). Dual occupation families and migration. American Sociological Review.

[CR26] Dustmann, C., & Glitz, A. (2011). Migration and education. In *Handbook of the economics of education* (pp. 327–439). Elsevier.

[CR27] Dyson T (2011). The role of the demographic transition in the process of urbanization. Population and Development Review.

[CR28] Field E, Molitor V, Schoonbroodt A, Tertilt M (2016). Gender gaps in completed fertility. Journal of Demographic Economics.

[CR29] Fleury, A. (2016). Understanding women and migration: A literature review. *KNOMAD Working Paper Series*, *February*, 48. https://www.knomad.org/publication/understanding-women-and-migration-literature-review-annex-annotated-bibliography

[CR30] Forste R (2002). Where are all the men? A conceptual analysis of the role of men in family formation. Journal of Family Issues.

[CR31] Geist C, McManus PA (2012). Different reasons, different results: Implications of migration by gender and family status. Demography.

[CR32] Goldberg D (1959). The fertility of two-generation urbanites. Population Studies.

[CR33] Goldstein S (1973). Interrelations between migration and fertility in Thailand. Demography.

[CR34] Gyimah SO (2006). Migration and fertility behavior in Sub-Saharan Africa: The case of Ghana. Journal of Comparative Family Studies.

[CR35] Hervitz HM (1985). Selectivity, adaptation, or disruption ? A comparison of alternative hypotheses on the effects of migration on fertility: The case of Brazil. The International Migration Review.

[CR36] Jensen ER, Ahlburg DA (2004). Why does migration decrease fertility?. Evidence from the Philippines. Population Studies.

[CR37] Joyner K, Peters HE, Hynes K, Sikora A, Taber JR, Rendall MS (2012). The quality of male fertility data in major U.S. surveys. Demography.

[CR38] King R, Skeldon R (2010). ‘Mind the Gap!’ Integrating approaches to internal and international migration ‘mind the gap!’ Integrating approaches to internal and international migration. Journal of Ethnic and Migration Studies.

[CR39] Kravdal Ø, Rindfuss RR (2008). Changing relationships between education and fertility: A study of women and men born 1940 to 1964. American Sociological Review.

[CR40] Kulu H (2008). Fertility and spatial mobility in the life course: Evidence from Austria. Environment and Planning A.

[CR41] Kulu H (2013). Expliquer la variation urbano-rurale de la fécondité. Regional Studies.

[CR42] Lee BS, Pol LG (1993). The influence of rural-urban migration on migrants’ fertility in Korea, Mexico and Cameroon. Population Research and Policy Review.

[CR43] Lerch M (2018). Fertility decline in urban and rural areas of developing countries. Population and Development Review.

[CR44] Lerch M (2019). Regional variations in the rural-urban fertility gradient in the global South. PLoS ONE.

[CR45] Levira, F., Todd, J., & Masanja, H. (2014). Coming home to die? The association between migration and mortality in rural Tanzania before and after ART scale-up. *Global Health Action*, *7*(Suppl.1). 10.3402/gha.v7.22956 10.3402/gha.v7.22956 PMC4032507 24857612

[CR46] Menashe-Oren A, Bocquier P (2021). Urbanization is no longer driven by migration in low- and middle-income countries (1985–2015). Population and Development Review.

[CR47] Menashe-Oren A, Stecklov G (2018). Rural-urban population age and sex composition in Sub-Saharan Africa. Population and Development Review.

[CR48] Montgomery MR, Casterline JB (1993). The diffusion of fertility control in Taiwan: Evidence from pooled cross-section time- series models. Population Studies.

[CR49] Montgomery, M. R., Stren, R., Cohen, B., & Reed, H. E. (2003). *Cities transformed: Demographic change and its implications in the developing world*. The National Academies Press.

[CR51] Omondi CO, Ayiemba EHO (2003). Migration and fertility relationship: A case study of Kenya. African Population Studies.

[CR52] Ortensi LE (2015). Engendering the fertility/migration nexus: The role of women’s migratory patterns in the analysis of fertility after migration. Demographic Research.

[CR53] Pongi Nyuba, R. (2019). *Migration et fécondité en Afrique subsaharienne*. Université Catolique de Louvain.

[CR54] Potts D (1995). Shall we go home? Increasing urban poverty in African cities and migration processes. Geographical Journal.

[CR55] Potts D (2009). The slowing of sub-Saharan Africa’s urbanization: Evidence and implications for urban livelihoods. Environment and Urbanization.

[CR56] Ratcliffe AA, Hill AG, Dibba M, Walraven G, Ratcffe AA, Hid AG, Dibba M, Walraven G (2001). The ignored role of men in fertility awareness and regulation in Africa. Journal of Reproductive Health.

[CR57] Rogers A, Raymer J, Willekens F (2002). Capturing the age and spatial structures of migration. Environment and Planning A.

[CR58] Schoumaker B (2017). Measuring male fertility rates in developing countries with demographic and health Surveys: An assessment of three methods. Demographic Research.

[CR59] Schoumaker, B. (2019). Male fertility around the world and over time: How different is it from female fertility? *Population and Development Review*, 459–487. 10.1111/padr.12273

[CR60] Schoumaker, B., & Sánchez-Páez, D. A. (2020). Identifying fertility stalls by place of residence in sub-Saharan Africa. *PAA Annual Meeting*.

[CR61] Shapiro, D., & Gebreselassie, T. (2008). Fertility transition in Sub-Saharan Africa: Falling and stalling. *Étude de la Population Africaine*, *23*(1), 3–23. 10.11564/23-1-310

[CR62] Shapiro, D., & Tambashe, O. (1999). *Fertility transition in urban and rural areas of sub-Saharan Africa*. Population Research Institute, Pennsylvania State University. http://www.econ.psu.edu/~dshapiro/Chaire_Quetelet_paper.pdf

[CR63] Shapiro D, Tenikue M (2017). Women’s education, infant and child mortality, and fertility decline in rural and urban sub-Saharan Africa. Demographic Research.

[CR64] Smith-Greenaway E, Trinitapoli J (2014). Polygynous contexts, family structure, and infant mortality in sub-Saharan Africa. Demography.

[CR65] Stecklov, G., & Menashe Oren, A. (2019). *The demography of rural youth in developing countries* (No. 41; 2019 Rural Development Report Background Papers).

[CR66] Thomas MJ (2019). Employment, education, and family: Revealing the motives behind internal migration in Great Britain. Population, Space and Place.

[CR67] Timæus IM, Reynar A (1998). Polygynists and their wives in Sub-Saharan Africa: An analysis of five demographic and health surveys. Population Studies.

[CR68] Tragaki A, Bagavos C (2014). Male fertility in greece: Trends and differentials by education level and employment status. Demographic Research.

[CR69] United Nations. (2009). Overcoming barriers: Human mobility and development. In (UNDP) United Nations Development Programme (Ed.), *Human development report*. Palgrave Macmillan.

[CR70] United Nations. (2019). *World fertility data*. https://www.un.org/development/desa/pd/data/world-fertility-data

[CR71] Verkroost, F. C. J., & Monden, C. W. S. (2022). Childlessness and development in Sub-Saharan Africa: Is there evidence for a U-shaped pattern? *European Journal of Population, 38*(3), 319–352. 10.1007/s10680-022-09608-5 10.1007/s10680-022-09608-5 PMC9363553 35966357

[CR72] Yabiku ST, Agadjanian V, Sevoyan A (2010). Husbands’ labour migration and wives’ autonomy, Mozambique 2000–2006. Population Studies.

